# Prospective Evaluation of Early Complications After Elective Loop Ileostomy: Need to Optimise Loperamide Management?

**DOI:** 10.1007/s11605-021-05148-1

**Published:** 2021-09-24

**Authors:** Bolckmans Roel, Cornille Jean-Baptiste, Singh Sandeep, Boyce Stephen, D’hoore André, Wolthuis Albert

**Affiliations:** 1 Department of Colorectal Surgery, NIHR Oxford Biomedical Research Centre, Oxford University Hospitals NHS Foundation Trust, Oxford, UK; 2 Department of Colorectal Surgery, University Hospital Gasthuisberg, Leuven, Belgium

**Keywords:** Loop ileostomy, Complications, Readmissions, Stoma output, Loperamide

## Introduction

The need for a standard diverting ileostomy during “low” colorectal resections is continuously questioned because of increased awareness of the associated morbidity, with a move towards a “selective” use and close postoperative follow-up (FU).^1^

Most data in literature regarding ileostomy-related complication originates from studies with a retrospective design, with its known associated weaknesses of data incompleteness and lack of detailed recording.^2^ The aim of our study was to assess loop ileostomy-related complications in a prospective way, with focus on ileostomy output.

## Materials and Methods

Prospective data collection between October 2017 and August 2018 in two high volume colorectal centres. All patients who underwent an elective loop ileostomy formation were included.

Distance from the ileocecal valve and surgical technique was applied as per surgeons’ preference, and loperamide management was not standardised. All FU data were recorded during in-person clinic visits. Photographs were taken of patients’ loop ileostomy after their written consent at standardised time points during the study, to reassure an objective assessment over the two centres.

Means ± standard deviation or median with interquartile range was used according to distribution, T-test, or Mann–Whitney U test to compare numerical data and Chi-square or Fisher’s exact for categorical data. *P* ≤ 0.05 was considered as the significance level.

## Results

Mean hospital stay of the 81 included patients was 11 ± 9 days. None of the patients had a history of/or concomitant small bowel resection.

No ileostomy-related reinterventions were recorded during admission. Mean stoma output at discharge was 696 ± 412 ml/day, with 27/81 (33%) being on loperamide (Table 1). Twenty-six patients (32%) had an high output stoma (HOS, ≥ 1500 ml/day) during admission, with a significantly higher mean stoma output of 854 ± 386 ml/day at time of discharge (*p* = 0.03). There was a delay in discharge due to stoma-related issues in 17 patients (21%); because of HOS in 9 and stoma care in 8 patients (stoma training/application problems).

**Table 1:** Ileostomy complications during admission, 1-month and 3-month clinical follow-up

	Admission (*N* = 81)	One-month (56/74 (76%)) ^a^	Three-month (53/69 (77%)) ^b^
Daily loperamide N (%)	27 (33)	13 (23)	16 (30)
Stoma output (median number of emptied stoma bags a day)	^(f)^	5 (IQR 4–6)	5 (IQR 4–6)
Necrosis N (%) ^c^	3 (4)	NA	NA
Hematoma with clinical impact ^d^ N (%)	0 (0)	NA	NA
Peristomal abscess/cellulitis N ^e^ (%)	2 (3)	0 (0)	0 (0)
Nocturnal emptying N (%)	NA	40 (71)	42 (79)
Dermatitis N (%)	NA	20 (36)	10 (19)
Mucocutaneous separation N (%)	3 (4)	4 (7)	0 (0)
Retraction stoma N (%) Flush N (%)	2 (2) 4 (5)	5 (9) 6 (11)	1 (2) 9 (17)
Stoma bag leaks N (%) If a leak, the median number of stoma bag leaks	31 (38) 2 (IQR 1–3)	20 (36) 1 (IQR 1–5)	19 (36) 1 (IQR 1–2)
Number of Readmissions	NA	-HOS: 2 -LOS: 3 - Loperamide: 1 - (Sub)obstruction: 2 -Parastomal infection: 1	-HOS:1 -LOS: 3 - (Sub)obstruction: 3 -Stoma bleeding: 2

*N* number of patients, *IQR* interquartile range, *NA* not applicable, *HOS* high output stoma, *LOS* low output stoma

Admission data were evaluated and reported at the time of discharge

^a^75 patients hospital stay < 4 weeks with one patient deceased 3 weeks after surgery (subarachnoid bleeding)

^b^69 patients still alive and not reversed three months after index surgery

^c^Defined as the necrotic appearance of ileostomy mucosa (all managed successfully conservatively, no need for reintervention, BMI patients 23.3 / 26.4 / 30.6)

^d^Two patients on therapeutic anticoagulation before surgery who didn’t experience postoperative hematoma

^e^Defined as the need for antibiotic therapy for peristomal cellulitis

^f^Detailed recording in ml/day

Median loperamide dose after 1 month 5 mg (3–8), after 3 months 6 mg (4–12)

Admission data were evaluated and reported at the time of discharge

At 3-month FU, 79% reported nocturnal emptying with 36% of them being on loperamide therapy, 36% experienced stoma bag leaks.

Two patients (2.5%) had an early ileostomy closure because of related morbidity (stoma bag leaks, peristomal fistula).

There were 12 readmissions in 8 patients (10%) because of ileostomy-related complications, with in 4/8 patients (50%) because of ileostomy output-related complications and need for intensive care unit admission in one patient within a week after discharge (stoma output at discharge 975 ml/day) (Table 1).

## Discussion

HOS-related complications together with postoperative ileus/obstruction and deep infections are seen as the main reasons for readmission after elective colorectal surgery.^3^ The results of our study confirm that morbidity related to the first factor is significant, with HOS-related readmission in three patients (4%) and need for intensive care unit admission in one of them within 1 week after discharge. Additionally, 9 patients (11%) experienced a delay in discharge during elective admission because of HOS which is a known risk factor for readmission.^4^

**Fig. 1:**
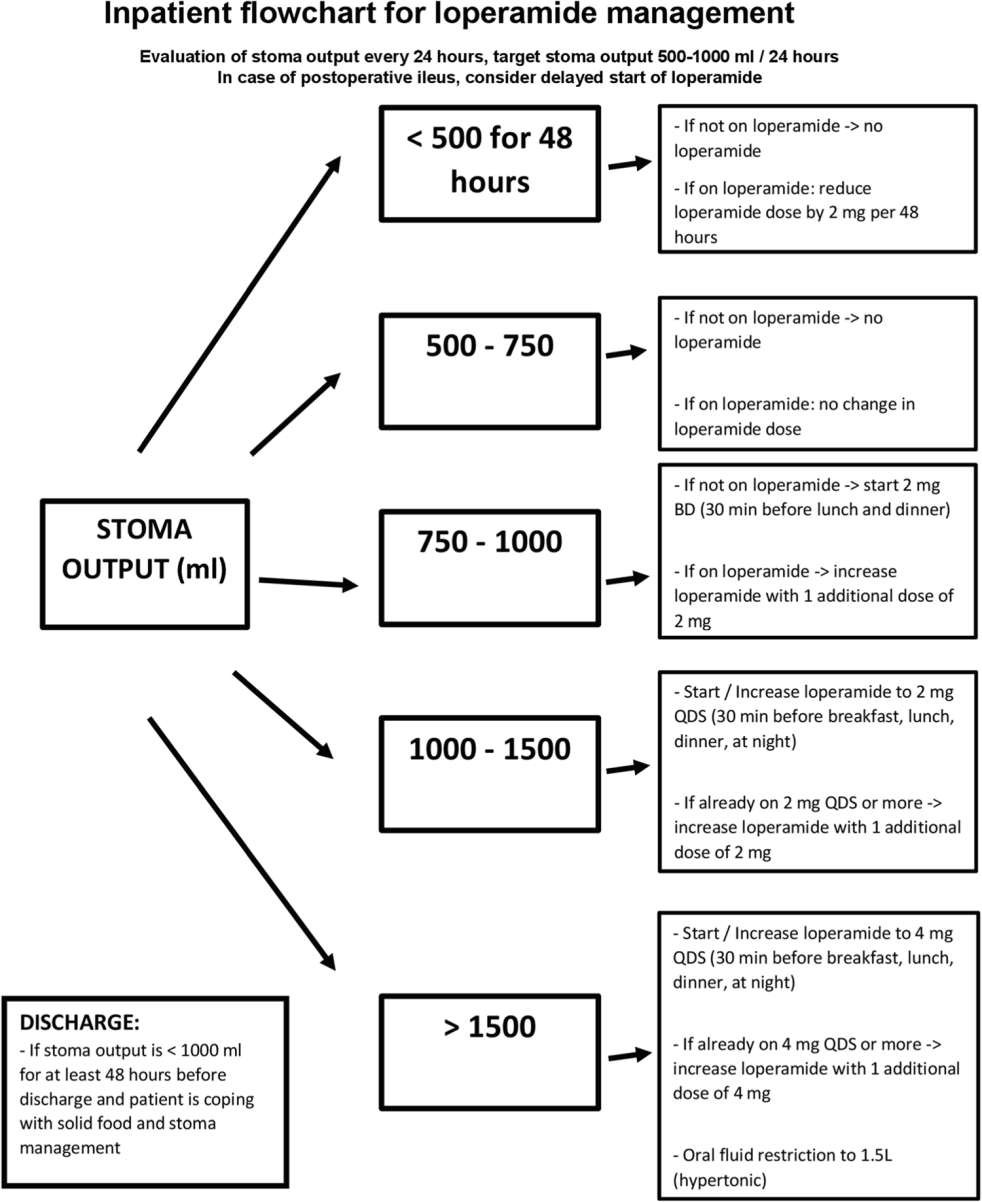
Inpatient flowchart

With those observations in mind, our centres put forward a flowchart to aim for consistent stoma output management during admission and discharge with a focus on loperamide therapy [Fig. 1]. In our opinion, this will result in a more systematic approach in ileostomy output management.

The importance of careful management of patients’ ileostomy output is further supported by a possible impact on their long-term kidney function.^5^

Strength of our study is the prospective design which allowed a detailed assessment of the ileostomy-related measures and a weakness the limited number of patients.

Further research has to focus on the question if more consistent use of loperamide therapy during admission and FU can further reduce readmission rates and morbidity associated with ileostomy formation.
